# Hemoglobin, albumin, lymphocytes and platelets (HALP) score as a predictor of survival in patients with glioblastoma (GBM)

**DOI:** 10.1186/s12883-024-03639-7

**Published:** 2024-07-26

**Authors:** Ozden Demir, Guzin Demirag, Furkan Cakmak, Demet Işık Bayraktar, Leman Tokmak

**Affiliations:** 1 Department of Medical Oncology, Faculty of Medicine, Ondokuz Mayıs University, Samsun, Turkey; 2 Department of Internal Medicine, Faculty of Medicine, Ondokuz Mayıs University, Samsun, Turkey; 3 Department of Biostatistics, Faculty of Medicine, Ondokuz Mayıs University, Samsun, Turkey

**Keywords:** Glioblastoma (GBM), HALP, Prognostic factor

## Abstract

**Background:**

We aimed to investigate whether the HALP score was a predictor of survival in patients with Glioblastoma (GBM).

**Methods:**

A total of 84 Glioblastoma (GBM) patients followed in our clinic were included in the study. HALP scores were calculated using the preoperative hemoglobin, albumin, lymphocyte and platelet results of the patients. For the HALP score, a cut-off value was found by examining the area below the receiver operating characteristic (ROC) curve. Patients were divided into two groups as low and high according to this cut-off value. The relationships among the clinical, dermographic and laboratory parameters of the patients were examined using these two groups.

**Results:**

Median OS, PFS, HALP score, NLR, PLR were 15 months (1.0–78.0), 8 months (1.0–66.0), 37.39 ± 23.84 (min 6.00-max 132.31), 4.14, 145.07 respectively. A statistically significant correlation was found between HALP score and OS, PFS, NLR, PLR, ECOG-PS status using Spearman’s rho test (*p* = 0.001, *p* < 0.001, *p* < 0.001, *p* < 0.001, *p* = 0.026 respectively). For the HALP score, a cut-off value of = 37.39 (AUC = 0.698, 95% CI, *p* < 0.002) was found using ROC analysis. Median OS was 12 (6.99–17.01) months in the low HALP group and 21 (11.37–30.63) months in the high HALP group (*p* = 0.117). NLR and PLR were significantly lower in the HALP high group (*p* < 0.001, *p* < 0.001 respectively). The ratio of receiving treatment was significantly higher in the high HALP group (*p* < 0.05). In Multivariate analysis, significant results were found for treatment status and ECOG-PS status (*p* < 0.001, *p* = 0.038 respectively).

**Conclusions:**

The HALP score measured at the beginning of treatment seems to have predictive importance in the prognosis of GBM patients. A HALP score of > 37.39 was associated with prolonged survival in high-grade brain tumors.

## Background

Glioblastoma (GBM) is the most common primary malignant brain tumor in adults. It usually has a poor prognosis. The standard treatment for newly diagnosed patients is first maximal surgery followed by the Stupp protocol. In the Stupp protocol, after surgery, first CCRT (Concurrent Chemoradiotherapy) is applied, followed by chemotherapy (CT) [1]. Although survival is improved with these treatments, most patients relapse. Median overall survival is approximately 15 months. Even most of the patients who receive maximum treatment die within two years [2, 3]. Although numerous studies have been conducted on this disease, limited progress has been achieved to improve the poor survival of it [4]. Prognostic factors affecting survival include age, Karnofsky performance score, chemotherapy administration, total radiation dose, location of the tumor in the brain, and complete tumor resection [5–16]. However, there are still not enough biomarkers to be used to follow patients and predict prognosis after GBM diagnosis.

Gliomas are classified as low-grade and high-grade according to their histological features. GBM includes high-grade (grade 3 and grade 4 anaplastic infiltrative gliomas) gliomas [17]. Molecular classification is also of great importance in the diagnosis of GBM. Molecular classification does not change the grading, but instead helps predict prognosis and guide treatment selection. Depending on the mutation status of the isocitrate dehydrogenase (IDH) gene, GBM may be IDH wild type or IDH mutant. IDH mutation in GBM is frequently associated with TP53 mutation. In general, IDH mutant GBM has a better prognosis than IDH-wild-type glioblastoma [18]. O6-methylguanine-DNA methyltransferase (MGMT) is a DNA repair enzyme. Methylation of this gene responds well to temozolomide (TMZ) treatment in GBM and is associated with better overall survival [18]. Other common molecular genetic alterations associated with GBM: TERT gene mutations, phosphatase and tensin homolog (PTEN) mutations, epidermal growth factor receptor (EGFR) amplification, cyclin-dependent kinase 4 (CDK4) amplifications, and cyclin-dependent kinase inhibitor 2 A (CDKN2-A) homozygous deletion [19]. In general, GBM is highly heterogeneous in terms of their molecular structure, making it difficult to find the best treatment.

It has been reported in studies conducted on many types of cancer that hemoglobin, albumin, lymphocyte, platelet (HALP) score can be a new prognostic predictor [20–30]. Although this combination has been studied for many cancer types, there are still not enough studies for GBM. The two most common indices, hemoglobin and albumin, reflect the nutritional status and performance of patients [31]. Since oxygen transport to the tumor tissue decreases in case of anemia, changes occur in some gene expressions and proteomic factors (e.g., vascular endothelial growth factor, epidermal growth factor, erythropoietin, glucose transporters and glycolytic enzymes). This catalyzes tumor survival, proliferation and invasion into surrounding tissues, which results in a worse prognosis for the patient [32, 33]. Malnutrition in cancer patients causes low albumin levels. Albumin not only shows the nutritional status but also acts as a carrier and antioxidant in the body. Since albumin is a negative acute phase protein, its decrease may indicate an increase in inflammation in the body [34]. Lymphocytes are involved in the recognition of tumor cells and indirectly in inhibiting and killing tumor cells [35]. Thrombocytes induce angiogenesis as follows: Thrombocyte releases pro-angiogenic factors and angiogenesis inhibitors, growth factors and some proteolytic enzymes, ultimately contributing to angiogenesis during tumor development and metastasis [36]. In short, while the increase in hemoglobin, albumin and lymphocyte contributes positively to the prognosis in cancer patients, the increase in thrombocyte contributes negatively. Albumin-based indices, fibrinogen-albumin ratio (FAR), neutrophil-lymphocyte ratio (NLR), platelet-lymphocyte ratio (PLR), and neutrophil, lymphocyte, platelet percentages alone are used to predict prognosis in several cancer types [37, 38].

HALP score appears to be a novel composite marker which can be easily tested in clinical practice, indicating both nutritional and inflammatory status of cancer patients. HALP score correlates positively with the prognosis of most cancers [39–42]. However, to the best of our knowledge, there is only one study in literature conducted with fewer cases on GBM. In this study by Korkmaz M et al., 31 patients who received bevacizumab + irinotecan for recurrent GBM were evaluated. HALP cut-off value was found to be 18. Patients were divided into two groups: under 18 and over. OS was found to be statistically significantly higher in the high HALP score group (9.63 [7.28–11.9]) compared to the low HALP score group (2.26 [0.88–3.65]) (*p* < 0.001). In univariate analysis, HALP score was shown to be a significant prognostic factor. The prognosis of patients with low HALP scores was found to be worse than those with high HALP scores (HR: 0.063, *p* < 0.001) [43]. The aim of this study is to demonstrate the predictive importance of the HALP score in the prognosis of patients diagnosed with GBM.

## Material method

A total of 84 patients diagnosed at Ondokuz Mayıs University, Faculty of Medicine between January 2015 and January 2021 were included in the present study. The diagnosis of Glioblastoma (GBM) for all patients was confirmed by the postoperative pathology report. The diagnosis of GBM was made by at least 2 expert pathologists, one of whom was a neuropathologist. 2021 WHO criteria were used for patients diagnosed in 2021 and later, and 2016 WHO diagnostic criteria were used for patients diagnosed before 2021. GBM was diagnosed histopathologically. However, molecular staining and immunohistochemical staining were performed in some patients. The study protocol was approved by Ondokuz Mayıs University, Ethics Committee. Inclusion criteria for the study were being older than 17 years of age, having no other diagnosis of malignancy or acute renal failure, acute liver failure, acute infection, and acute heart disease.

Serum albumin, hemoglobin, platelet, lymphocyte, neutrophil and many other blood test results of the patients were obtained before the start of treatment and before surgery. In addition, Isocitrate dehydrogenase (IDH) mutation, p53 mutation, and KI67 values ​​were obtained from the pathology report. Inflammatory indices were calculated with the following formulas: NLR = neutrophil count/lymphocyte count; PLR = platelet count/lymphocyte count; [5]. HALP score was calculated with the following formula: hemoglobin (g/L) × albumin (g/L) levels × lymphocyte count (/L)/platelet count (/L) [21]. The performance score of the patients was calculated according to the ECOG-PS. OS was calculated as the time from diagnosis to death or the last visit date. The primary endpoint was defined as OS. A cut-off value for the HALP score was found using Roc analysis according to the patients’ OS duration. Patients were divided into two groups as low and high according to this cut-off value. Each group was examined within itself regarding OS, PFS, NLR, PLR, ECOG-PS, p53 mutation, IDH mutation, residual status, tumor location, age and gender.

### Statistical analysis

Statistical analyses were performed with SPSS 21.0 for windows. Data were presented as mean ± standard deviation (SD), as median (min-max) as frequency (%). The Shapiro–Wilk test was used to analyze normal distribution assumption of the quantitative outcomes Data were analyzed with Student *t*-test and Mann–Whitney test for normal and non-normal data respectively. Results were evaluated using the nonparametric Kruskal–Wallis for comparison between groups. The frequencies were compared using the Pearson Chi-square and Continuity Correction Chi-square. The relation between variables was assessed by Spearman rank correlation for non-normal data. The area under the ROC curve (AUC) was evaluated as the measure of a diagnostic test’s discriminatory power. Confidence intervals can be computed for AUC. In this article, both sensitivity and specificity values were evaluated.

Kaplan-Meier method was used for survival analysis with the log-rank test used to statistical difference. HALP analysis and Kaplan Meier plot of survival are provided. A univariate Cox proportional hazards regression model was used to evaluate the prognostic value of each variable for OS. Multivariate Cox proportional hazards regression models were used to analyze independent prognostic factors. A p value less than 0.05 was considered as statistically significant.

## Results

A total of 84 patients were included in the study. Of these, 49 (58.3%) were male and 35 (41.7%) were female. Median age was 58 ± 15.06 (18–87) years. Median follow-up was 15.0 (1.0–78.0) months. Median PFS time was 8.0 (1.0–66.0) months. As for the ECOG-PS of the patients, 9 (10.7%) had 0, 32 (38.1%) had 1, 33 (39.3%) had 2 and 10 (11.9%) had 3. While 38 (45.2%) patients had a comorbid disease, the remaining 46 (54.8%) did not have any comorbid disease. All patients were operated on. Partial resection was performed in 69 (82.1%) patients and total resection was performed in 15 (17.9%) patients. None of the radiotherapy and/or chemotherapy treatments were applied to 19 (22.6%) patients after surgery. A total of 77 patients were investigated for IDH mutation. Of these, 62 (73.8%) did not have any IDH mutation (wild type) while 15 (17.9%) had IDH mutation. All patients were investigated for p53 mutation. As a result, it was found that 40 (47.6%) patients had this mutation while 44 (53.4%) did not.

The relationship between HALP score and age, OS, PFS, NLR, PLR, ECOG was examined using Spearman’s rho test. While there was a weak and positive correlation between HALP score and, OS and PFS (rho = 0.361, 0.381, *p* = 0.001, *p* < 0.001 respectively), a significant negative correlation was found between HALP score and, NLR, PLR and ECOG-PS status (rho = − 0.717(strong), -0.942(very strong), -0.243(weak), *p* < 0.001, *p* < 0.001, *p* = 0.026 respectively). The median HALP score in patients who received any of the CRT or Stupp protocol treatments after surgery was 38.41 (6.60-132.30). In patients who had surgery only, the median HALP score was 18.90 (6.00-56.60). The HALP score was significantly higher in patients who received either CRT or Stupp protocol after surgery (*p* < 0.001). The median HALP scores were 45.40 (6.60-132.30), 23.78 (6.00-71.10) for male and female patients respectively and there was a statistically significant difference between the two groups (*p* = 0.005).

**Fig. 1:**
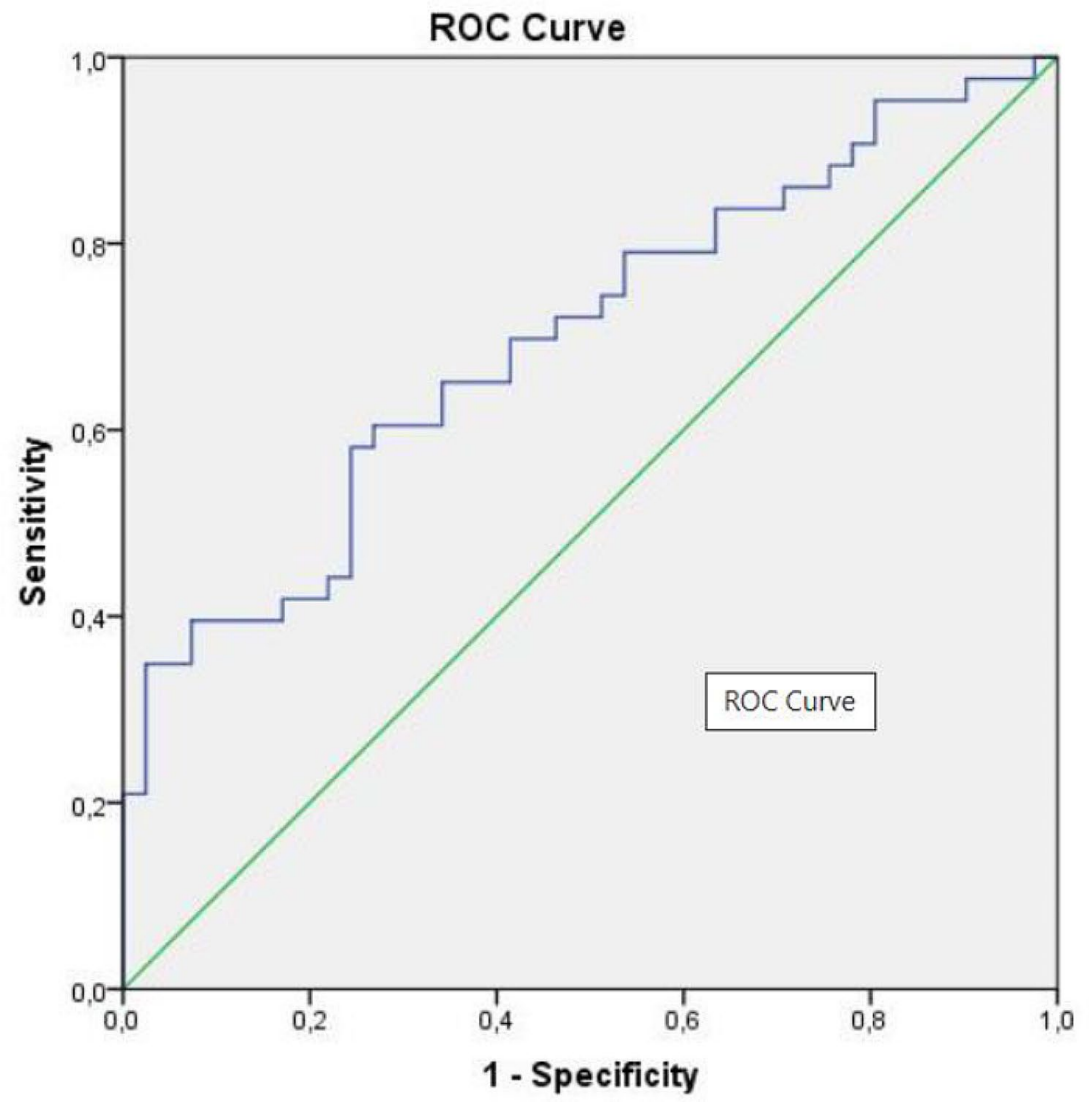
Halp score cut-off value with ROC analysis

Patients were divided into two groups as OS 15 months and over and, under 15 months. The median HALP score was 45.40 (6.60-132.31) for the 15 months and over group and 27.33 (6.00-66.5) in the group under 15 months which was statistically significantly high (*p* < 0.001). Median HALP score was 37.39 ± 23.84 (6.00-132.31) for the entire patient population. The HALP score cut-off value was found as 37.39, using ROC analysis (AUC = 0.698, 95% CI, 0.587–0.810 *p* = 0.002) (Fig. 1).

While the median OS of Group 1 patients who had a cut-off value for HALP score under 37.39, according to which the present study had been planned, was 12 (95%, 6.99–17.01) months, median OS was 21 (95% CI, 11.37–30.63) months for Group 2 who had high HALP scores. There was no statistically significant difference (*p* = 0.117). Table 1 presents the comparison of HALP score according to several parameters. Median NLR and PLR were significantly lower in Group 2 compared with those of Group 1 (*p* < 0.001 and *p* < 0.001 respectively). It was found that NLR and PLR parameters decreased as HALP score increased. HALP scores measured before treatment were significantly higher in patients who underwent CRT or Stupp protocol after surgery than in patients who underwent surgery alone (*p* = 0.010). Fifteen (36.6%) of 19 (22.6%) patients who did not receive any treatment after surgery had low HALP scores. In the group with a high HALP score, only 4 (9.3%) patients did not receive any treatment after surgery. The median age was observed to be lower in Group 2. There was a significant relationship between high HALP scores and age (*p* = 0.001) (Table 1).

With Kaplan Meier analysis, the relationship between OS and treatment status, ECOG status, gender, p53 mutation, IDH mutation, residual tissue, comorbidity, tumor location, and surgery type was examined. The median OS was 2.5 (95%, 1.64–3.35) months in the surgery-only group while it was 20 (95%, 14.65–25.34) months in the group that received any post-surgical treatment (CRT or Stupp protocol). A statistically significant correlation was found between treatment status and OS (*p* < 0.001). Patients were divided into two groups as ECOG-PS 0–1 and 2–3. A significant relation was found between these two groups and OS (*p* = 0.003). Those with ECOG-PS 0–1 had significantly longer survival than those with 2–3 (median OS 20 months-9 months respectively). No significant correlation was found between OS and, gender, IDH mutation, p53 mutation, residual, additional disease, type of surgery and disease location.

The effects of the patients’ clinical, laboratory and dermographic data on survival were analyzed with Cox Regression analysis. Univariate analysis showed that treatment status (surgery -only or CRT / Stupp protocol), age (a per unit increase), and ECOG-PS (0 vs. 3) were important prognostic factors (Table 2). The risk increased 1.022 times with one unit increase in age. This was statistically significant (*p* = 0.018). The cut-off value found for HALP score was not significant in the univariate analysis. Multivariate analysis showed that treatment status (*p* < 0.001) and ECOG-PS (*p* = 0.038) were important prognostic factors for OS. Hazard Ratio (HR) values ​​of ECOG 1 and 2 were 1.128 and 1.166 respectively compared with ECOG 0, and these values ​​were statistically insignificant (*p* = 0.793 and *p* = 0.750). However, the HR value of ECOG 3 was 3.181 compared with ECOG 0, *p* = 0,038. Namely, death risk for ECOG 3 was 3.181 times higher than that of ECOG 0. HR value of the untreated patients was 13.640 compared with the treated patients, *p* < 0,001. In other words, the risk of death in patients who had surgery-only was 13,640 times higher than in patients who received either CRT or Stupp protocol treatments after surgery, and this was statistically significant (Table 2).

**Fig. 2:**
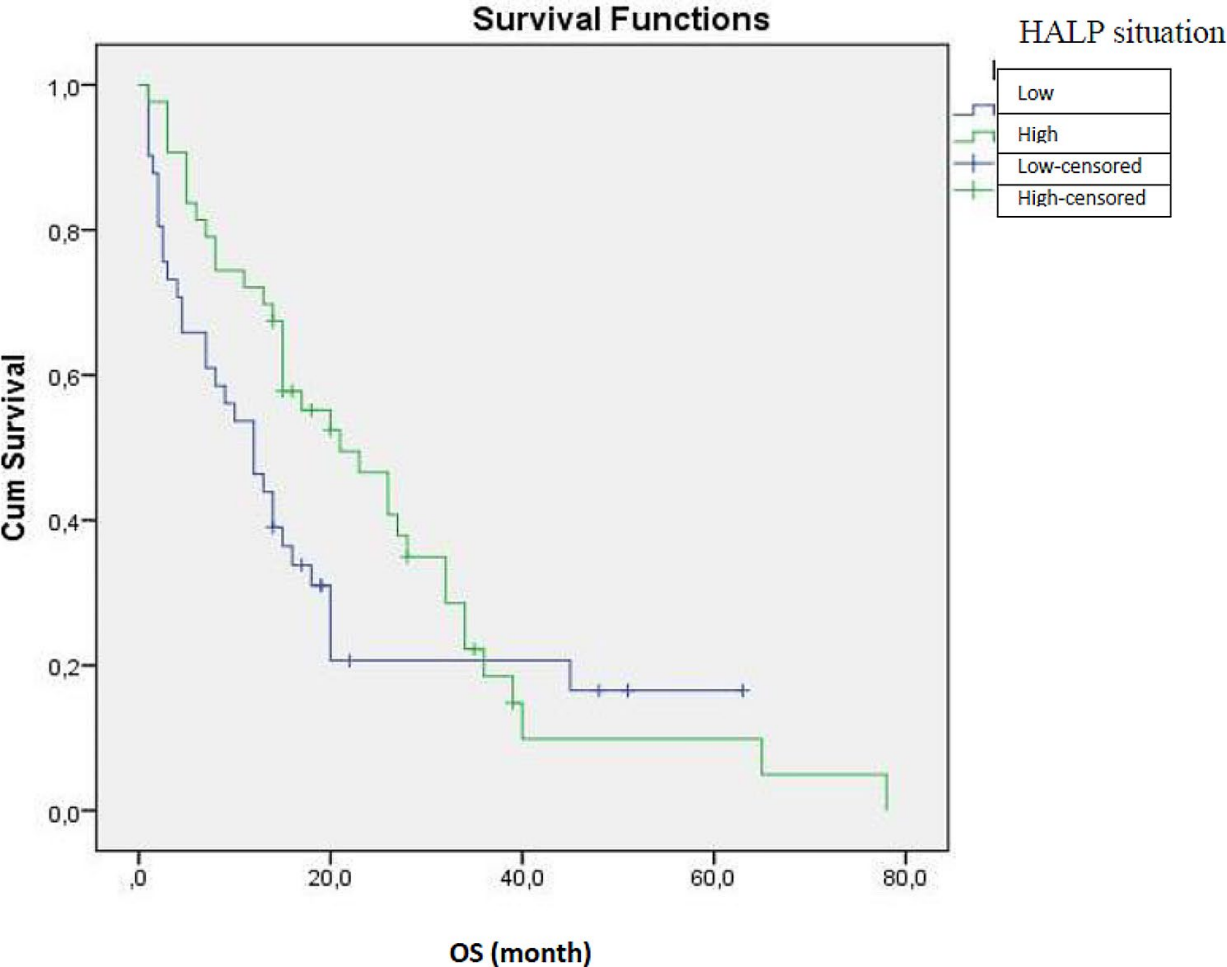
HALP analysis and Kaplan Meier plot of survival

HALP analysis and Kaplan Meier plot of survival are provided. The median survival value is 12 (6.99–17.01) for the HALP low group and 21 (11.37–30.63) for the high group. The two groups were compared in terms of survival, it was found that there was no statistical difference between them (*p* = 0.117) **(**Fig. 2**)**.

**Table 1:** Demographic, clinical and laboratory characteristics of the patients according to the HALP score

Features	Total (n:84)	HALP Score		
		< 37.39 (n:41)	≥ 37.39 (n:43)	p value
**Age (years) (mean ± sd.)**		61.07 ± 14.24	53.44 ± 15.04	**0.019**
**Gender (n [%])**				**0.051**
**Male**	49 (%58,3)	19(46.3%)	30(69.8%)	
**Female**	35 [7, 40]	22(53.7%)	13(30.2%)	
**ECOG-PS (n [%])**				0.222
**0**	9(10.7%)	2(4.9%)	7(16.3%)	
**1**	32 (38.1%)	14(34.1%)	18(41.9%)	
**2**	33(39.3%)	19(46.3%)	14(32.6%)	
**3**	10(11.9%)	6(14.6%)	4(9.3%)	
**Residual tumor (n [%])**				0.919
**No**	15 (%17,9)	8(19.5%)	7(16.3%)	
**Yes**	69 (%82,1)	33(80.5%)	36(83.7%)	
**Comorbid disease (n [%])**				0.083
**Yes**	38(45.2%)	23(56.1%)	15(34.9%)	
**No**	46(54.8%)	18(43.9%)	28(65.1%)	
**Treatment status (n [%])**				**0.010**
**Only surgery**	19(22.6%)	15(36.6%)	4(9.3%)	
**Surgery + CRT**	8(9.5%)	4(9.8%)	4(9.3%)	
**Surgery + Stupp Protocol ( CCRT-> CT )**	57(67.8%)	22(53.7%)	35(81.4%)	
**Surgery Type (n [%])**				0.919
**Full resection**	15(17.9%)	8(19.5%)	7(16.3%)	
**Partial resection**	69(82.1%)	33(80.5%)	36(83.7%)	
**PFS (month) (median + min-max)**		4.0(1.0–51.0)	10.0(1.0–66.0)	
**OS (month) (median + 95%CI low/up)**		12 (6.99–17.01)	21 (11.37–30.63)	0.117
**NLR (median + min-max)**		7.08(1.4–29.1)	2.69(1.29–8.06)	**< 0,001**
**PLR (median + min-max)**		238.8(66.5-757.1	110.40(50.9-160.4)	**< 0,001**
**Tumor location (n [%])**				0.904
**Frontal**	22(26.2%)	11 (26.8%)	11 (25.6%)	
**Parietal**	22 (26.2%)	12 (29.3%)	10 (23.3%)	
**Temporal**	22 (26.2%)	11 (26.8%)	11 (25.6%)	
**Occipital**	8 (9.5%)	3 (7.3%)	5 (11.6%)	
**Others**	10 (11.9%)	4 (9.8%)	6 (14.0%)	
**P53 mutation (n [%])**				0.670
**Yes**	40(47.6%)	21(51.2%)	19(44.2%)	
**No**	44(52.4%)	20(48.8%)	24(55.8%)	
**IDH mutation (n [%])**				0.955
**Yes**	15(19.5%)	7(17.9%)	8(21.1%)	
**No**	62(80.5%)	32(82.1%)	30(78.9%)	
**Ex status**				0.912
**Yes**	67(79.8)	32(78.0%)	35(81.4%)	
**No**	17(20.2)	9(22.0%)	8(18.6%)	

SD: Standard deviation, KT: Chemotherapy, RT: Radiotherapy, CRT: Chemoradiotherapy, CCRT: Concurrent Chemoradiotherapy, Stupp Protocol: After surgery, first CCRT and then CT

**Table 2:** Univariate and multivariate analysis results of OS in patients with Glioblastoma Multiforme

	Univariate Analyses			Multivariate Analyses		
	HR	95% CI	p value	HR	95% CI	p value
**HALP Score (< 37.39 vs. ≥ 37.39)**	0.680	0.41–1.11	0.126			
**Gender (F vs. M)**	1.178	0.71–1.93	0.518			
**Age**	1.022	1.00-1.04	**0.018**	1.015	0.99–1.03	0.145
**ECOG-PS**						
**0**			**0.007**			0.066
**1**	1.122	0.45–2.75	0.802	1.128	0.45–2.77	0.793
**2**	2.015	0.82–4.90	0.123	1.166	0.45–2.99	0.750
**3**	3.954	1.37–11.34	**0.011**	3.181	1.06–9.50	**0.038**
**Residual Tumor** **(yes vs. no)**	1.393	0.68–2.82	0.357			
**Treatment Status (surgery-only/ CRT or Stupp protocol )**	13.418	6.83–26.35	**< 0.001**	13.640	6.33–29.38	**< 0.001**
**Chronic Disease**	1.487	0.91–2.41	0.109			

HR: Hazard ratio, CI: Confidence interval, F: Female, M: Male

## Discussion

Pre-treatment HALP score seems to have predictive significance on the prognosis in GBM patients with high-grade brain tumors. In many recent studies, HALP score has been suggested as a decent prognostic determinant among other hematological and biochemical combination parameters for certain cancer types. This results from the fact that hemoglobin and albumin in this combination also provide information about the nutritional status of the patient [31].

In the present study, we found a relation between HALP score and, OS and PFS. We found that the survival time of the patients increased as the HALP score increased (median OS 4.5 months in the low HALP group, median OS 11 months in the high HALP group). Although it was not statistically significant, there was a quantitative OS difference between the two groups. We believe that more precise results can be obtained with studies including more cases. Korkmaz M. et al. conducted a study on 31 patients and found that OS was significantly different between low HALP (2.26 [0.88–3.65]) and high HALP groups (9.63 [7.28–11.9]) (*p* < 0.001). However, all of their patients were diagnosed with recurrent GBM who received irinotecan + bevacizumab treatment after relapse; that is, their subjects were a homogeneous group [43]. In the present study, on the other hand, some of the 84 patients did not receive any treatment while some of them received more than one treatment. The heterogeneity in the treatment of our patients may account for this result.

In the present study, it was found that NLR and PLR decreased as the HALP score increased and that these inflammatory indices increased as the HALP score decreased. There was a significant negative correlation between HALP and, NLR and PLR. The relation between OS and, NLR and PLR has been showed in several studies conducted on many types of cancer [37–44]. Since lymphocytes have an important role in fighting tumor cells, their decrease is a negative factor [35]. Neutrophils, which can activate inflammatory cells, particularly cancer cells, play an important role in the tumor microenvironment [45]. Inflammation leads to a decrease in serum albumin levels [46–48]. This causes an increase in microvascular permeability. The albumin distribution between the intra- and extra-vascular compartments changes accordingly. As a result, it causes a decrease in the albumin level in the plasma [49]. A decrease in lymphocytes and/or an increase in neutrophils results in an increase in NLR. Therefore, an increase in NLR is considered as a poor prognostic factor. An increase in thrombocytes increases PLR because thrombocytes contribute to tumor angiogenesis [36], which may make PLR a poor prognostic factor. Although NLR, PLR, albumin and hemoglobin are easily accessible and cost-effective tests, they are affected by many factors. For instance, steroid treatment, surgery, hypertension, cardiovascular diseases, autoimmune diseases, acute infections, acute kidney and liver disorders, and diagnosis of other cancer types may affect neutrophil, platelet, hemoglobin, albumin, and lymphocyte values [50]. Therefore, we examined all factors associated with systemic inflammation before the operation and before any treatment was initiated. In addition, we did not include patients with other cancer types, acute infections, acute liver, kidney and heart disorders in the present study.

The patients in the present study were of ECOG-PS 0,1,2, and 3. There was a significant negative correlation between HALP and ECOG-PS. As the performance of the patients deteriorated, the HALP score decreased. The nutrition of patients with poor performance deteriorated, which reduced albumin levels. As albumin, which is a negative acute phase reactant, decreases, systemic inflammation increases and survival is significantly affected [34]. It has been showed that nutrition and the inflammatory response play a central role in cancer patients [25–31, 51]. HALP scores of patients who received any of the CRT or Stupp Protocol treatments after surgery were significantly higher than those who did not receive them. The majority of patients who could not receive treatment after surgery had poor ECOG-PS and their general condition was not suitable for treatment. Their nutritional statuses were impaired. Accordingly, albumin levels of these patients were low. OS of these patients was also low.

In the present study, median HALP score was significantly higher in male patients. In a previous study, it was showed that there was a significant correlation between HALP and gender [39]. Their results revealed that HALP score was higher in male patients compared to those of female patients. They concluded that the difference in hemoglobin levels between male and female patients might explain the significant relationship between gender and HALP score (female:127.1 g/L and male: 138.1 g/L, *P* < 0.001). However, there are studies in literature which did not find a significant relationship between gender and HALP [27, 29, 30, 52]. In a study of Feng JF et al., a significant correlation was found between gender and hemoglobin (114.2 ± 13.7 g/L for female and 117.8 ± 11.9 g/L for male, *p* = 0.033) [40]. In the present study, there was a significant relationship between gender and HALP score as well (*p* = 0.005).

Although HALP score has been investigated as a prognostic predictor for various cancers in literature, an optimal cut-off value has not been determined yet. In the only study in literature conducted on HALP in patients with GBM, the cut-off value was found using the X-tile software method [43]. In the present study, we determined the cut-off value for the HALP score by performing ROC curve analysis. Patients who were below this cut-off value formed the low HALP group. The mean age in this group was significantly higher. HALP score is a combination indicating the nutritional status of the patient. In the present study, it was observed that HALP score was lower in elderly patients. This may result from comorbid diseases and lower nutritional status of the elderly patients [53].

We think that there are many reasons why we could not find a statistically significant relationship between HALP and OS in our study. We know that there are many factors that affect survival. The more heterogeneous characteristics the patients included in the study have, the more different the survival results may be. The treatments our patients received after surgery were not the same. While some did not receive any treatment after surgery, some received only CRT and some received 1 or 2 series of CT after CRT. In the study conducted by Korkmaz M et al., all patients received the same treatments. This heterogeneity affects the OS times of patients. ECOG performances of the patients were distributed heterogeneously between 0 and 3. As we mentioned before, the HALP score was also found to be lower in low-performance patients. Those with an ECOG score between 1 and 2 were the patients whose OS was the most difficult to predict. The patients’ age, gender, presence of residual tumor, presence of comorbid diseases, IDH and p53 mutation status, and tumor location distributions were also heterogeneous. All these variables caused heterogeneous distribution. We think that the patient group with a heterogeneous distribution is the most important reason why we could not find a significant relationship between OS and HALP.

There were several limitations in the present study. First of all, our study was retrospective and prospective studies should be designed. Secondly, all patients were selected from a single center. Therefore, setting of the study should be taken into consideration when clinical results are interpreted. Therefore, multi-center studies involving more patients are required to confirm our findings. Another limitation of the present study was that HALP is an independent predictor for many tumors and has high levels of sensitivity and specificity.

## Conclusion

We believe that HALP score examined in GBM, which is a high-grade brain tumor, may be useful as a clinical prognostic factor. It was found that OS was lower in patients with low HALP scores and that it was higher in patients with high HALP scores. NLR and PLR parameters were also inversely and significantly correlated with the HALP score in patients with GBM. Survival was shorter in patients with high NLR and PLR. HALP score is an easily conducted and inexpensive test used to determine the prognosis and response to treatment in patients with GBM. Prospective studies involving more patients are required to demonstrate the predictive value of the HALP score.

## Data Availability

The datasets generated and/or analyzed during the current study are not publicly available due [Since there are hospital data] but are available from the corresponding author on reasonable request.

## Abbreviations

GBM: Glioblastoma
HALP: Hemoglobin, albumin, lymphocyte, and platelet score
NLR: Neutrophil-lymphocyte ratio
PLR: Platelet-lymphocyte ratio
ECOG-PS: Eastern Cooperative Oncology Group Performance Status
ROC: Receiver operating characteristic
AUC: Area Under Curve
CI: Confidence interval
OS: Overall survival
PFS: Progression free survival
RT: Radiotherapy
KT: Chemotherapy
FAR: Fibrinogen/albumin ratio
IDH: Isocitrate dehydrogenase mutation
SD: Standard deviation
HR: Hazard ratio
F: Female
M: Male
